# Machine learning in Huntington’s disease: exploring the Enroll-HD dataset for prognosis and driving capability prediction

**DOI:** 10.1186/s13023-023-02785-4

**Published:** 2023-07-27

**Authors:** Jasper Ouwerkerk, Stephanie Feleus, Kasper F. van der Zwaan, Yunlei Li, Marco Roos, Willeke M. C. van Roon-Mom, Susanne T. de Bot, Katherine J. Wolstencroft, Eleni Mina

**Affiliations:** 1 Department of Pathology and Clinical Bioinformatics, Erasmus Medical Center (EMC), Wytemaweg, 3015 CN Rotterdam, The Netherlands; 2 Department of Neurology, Leiden University Medical Center (LUMC), PO Box 9600, 2300 RC Leiden, The Netherlands; 3 Department of Clinical Epidemiology, Leiden University Medical Center (LUMC), PO Box 9600, 2300 RC Leiden, The Netherlands; 4 Department of Human Genetics, Leiden University Medical Center (LUMC), PO Box 9600, 2300 RC Leiden, The Netherlands; 5 Leiden Institute of Advanced Computer Science (LIACS), Leiden University, Niels Bohrweg 1, 2333 CA Leiden, The Netherlands

**Keywords:** Huntington’s disease, Enroll-HD, Data pre-processing, Machine learning, Recurrent neural networks

## Abstract

**Background:**

In biomedicine, machine learning (ML) has proven beneficial for the prognosis and diagnosis of different diseases, including cancer and neurodegenerative disorders. For rare diseases, however, the requirement for large datasets often prevents this approach. Huntington’s disease (HD) is a rare neurodegenerative disorder caused by a CAG repeat expansion in the coding region of the huntingtin gene. The world’s largest observational study for HD, Enroll-HD, describes over 21,000 participants. As such, Enroll-HD is amenable to ML methods. In this study, we pre-processed and imputed Enroll-HD with ML methods to maximise the inclusion of participants and variables. With this dataset we developed models to improve the prediction of the age at onset (AAO) and compared it to the well-established Langbehn formula. In addition, we used recurrent neural networks (RNNs) to demonstrate the utility of ML methods for longitudinal datasets, assessing driving capabilities by learning from previous participant assessments.

**Results:**

Simple pre-processing imputed around 42% of missing values in Enroll-HD. Also, 167 variables were retained as a result of imputing with ML. We found that multiple ML models were able to outperform the Langbehn formula. The best ML model (light gradient boosting machine) improved the prognosis of AAO compared to the Langbehn formula by 9.2%, based on root mean squared error in the test set. In addition, our ML model provides more accurate prognosis for a wider CAG repeat range compared to the Langbehn formula. Driving capability was predicted with an accuracy of 85.2%. The resulting pre-processing workflow and code to train the ML models are available to be used for related HD predictions at: https://github.com/JasperO98/hdml/tree/main.

**Conclusions:**

Our pre-processing workflow made it possible to resolve the missing values and include most participants and variables in Enroll-HD. We show the added value of a ML approach, which improved AAO predictions and allowed for the development of an advisory model that can assist clinicians and participants in estimating future driving capability.

**Supplementary Information:**

The online version contains supplementary material available at 10.1186/s13023-023-02785-4.

## Background

Huntington’s disease (HD) is a rare disease that causes progressive degeneration of the brain and is inherited in an autosomal dominant manner. On average, the age at onset (AAO) for HD is around 45 years [1]. HD causes involuntary movements, cognitive impairments, psychiatric and behavioural problems, and progressive weight loss [2]. The genetic cause of the disease is a CAG repeat expansion in the coding region of the huntingtin gene (*HTT*;ENSG00000197386), which is translated into an expanded stretch of glutamine amino acids in the huntingtin protein (HTT) [1]. This mutant protein is the main cause of neuropathology in HD [3]. Despite its discovery in 1993 [4], we still lack treatments that can prevent, delay, or cure the disease [5].

Enroll-HD [6] is a worldwide longitudinal study of HD that has collected observational data from HD patients for about 20 years. As a result, Enroll-HD has become a large and high quality dataset, describing over 20,000 participants of which 16,000 are Huntington’s disease gene expansion carriers. The Enroll-HD dataset provides cross-sectional and longitudinal observational information regarding HD patients’ symptoms, such as the CAG repeat length and family history of HD. In addition, Enroll-HD records information about participants’ nutrition, medication, medical history, as well as the assessments and care received at each visit to their healthcare providers. One of the latest versions of Enroll-HD (PDS5) includes 21,116 participants and 55,975 visits, where each participant can have up to 20+ healthcare visits [7]. Previous work on the application of machine learning (ML) models on Enroll-HD include evaluating the frequency and factors associated with psychosis in HD [8], predicting the development of suicidal ideas in HD patients [9], and predicting the size of the CAG expansion based on phenotypic data [10]. However, these studies used earlier versions (PDS2 or PDS3) of the Enroll-HD dataset, which described only around 4000–8000 participants. Also little information was provided regarding the pre-processing steps in each study. Another more recent study by Mohan et al. [11], used a probabilistic ML model to define stages of the disease. In addition, a study by Ghazaleh et al. [12] applied a random forest (RF) algorithm to identify the relative contribution of certain Enroll-HD variables to clinical disease progression. Another study by Ko et al. [13] applied K-Means clustering and trained a ML model (XGBoost) to predict the disease trajectory cluster. However, they still omitted details regarding the pre-processing steps and the Enroll-HD variables included in the analysis.

### Pre-processing enroll-HD

In this work, we focus on the importance of the pre-processing steps to maximise the number of participants and variables included in the analysis. Missing values, outliers and a lack of standardisation in data collection can hamper accurate predictions and must be addressed before any modelling and analysis. Here, we describe systematic and reproducible methods for pre-processing and imputation of missing values with ML. The pre-processed data can be used for additional studies, and future studies can reuse our workflow to pre-process and impute future releases of Enroll-HD in a similar fashion. We further demonstrate the utility of ML in HD for improving the estimation of the AAO and for continuous patient assessment using longitudinal data.

### Predicting the age at onset (AAO)

The AAO is one of the most important variables in the Enroll-HD dataset. It describes when an individual with a prolonged CAG-repeat in the HD causing range develops symptoms of the disease [14]. The length of the CAG repeat is inversely correlated to the AAO [15] and is the largest contributing factor for estimating the AAO [16]. Current models for AAO prediction use the length of the CAG repeat as the main predictor variable, which explains only around 70% of the observed variability in AAO [17]. Cases where HD patients have precisely the same CAG repeat length, but start to exhibit symptoms at different ages, indicate that there is more at play than just the CAG repeat. For example, additional variations in the genetic code between individuals affect the AAO, as demonstrated by studies on genetic modifiers of HD [18–20].

Currently, the Langbehn formula [15] is used to estimate the AAO, which is only based on the CAG repeat size. The formula was evaluated on CAG repeat sizes between 41 and 56. However, individuals can develop HD with a smaller or larger CAG repeat size. Therefore, there is a need for a model that can better explain the variability between the AAO and make better predictions regarding the AAO of Huntington’s disease gene expansion carriers. The accurate prediction of the AAO will become more important for upcoming clinical trials, which will investigate new (targeted) therapies to slow down disease progression, or even prevent disease onset. [21]. Here, more accurate AAO estimation could prevent unnecessarily starting therapy years too early. In this study, we aim to improve the AAO prediction, compared to the Langbehn formula, by training several multivariate ML models on a broader CAG repeat range.

### Assessing driving capability

Also, we explored the longitudinal properties of the Enroll-HD dataset and the advantages of using ML methods for continued assessment and monitoring. Currently, a patient’s driving capability is recorded in Enroll-HD as a binary indicator. However, in a complex neurodegenerative disease like HD, with motor, cognitive, and behavioural factors, which can all influence the driving capability over time, it is hard to estimate when an individual’s driving capability will be impaired. Making use of ML models involving all these aspects, that can learn from previous assessments to improve current and future assessments, could help clinicians in advising their patients. Here, we applied Recurrent Neural Networks (RNNs) on the longitudinal Enroll-HD dataset to learn from previous assessments and provide a personalised and future trajectory on driving capability.

## Methods

**Fig. 1:**
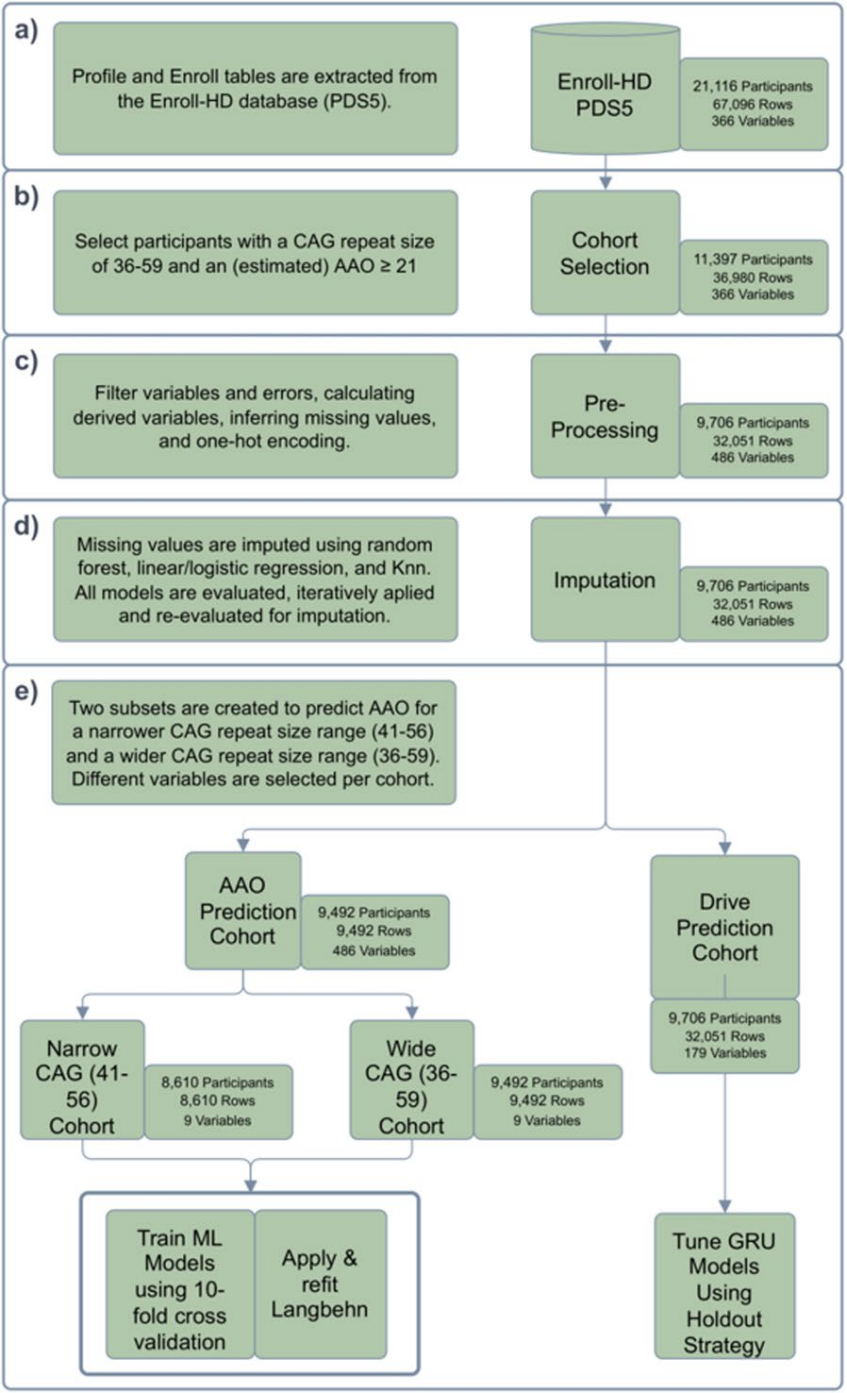
The workflow of the cohort selection, pre-processing, imputation, and ML model training steps. **a** Selected version and tables of Enroll-HD. **b** Inclusion criteria of our study. **c** Pre-processing steps for the reduction of the number of missing values in the cohort. **d** Imputation of the remaining missing values using ML models. **e** Prediction steps. For the AAO prediction two cohorts are created one for the narrow CAG size (41–56) and another for the wide CAG size (36–59) to fit and evaluate the ML models and the Langbehn formula. For the driving capability the GRUs are fitted and evaluated with multiple hyperparameters

Here we describe the steps required to pre-process Enroll-HD and to train ML models for the prediction of the AAO and driving capability. Firstly, the inclusion criteria, pre-processing steps, and the imputation of missing data is explained. After these steps the development of the ML models for AAO prediction and assessing the driving capability is described. The complete workflow is shown in Fig. 1 and described below. A more detailed workflow is available in Additional file 1.

### Cohort

Data used in this work were generously provided by the participants in the Enroll-HD study and made available by CHDI Foundation, Inc. Enroll-HD is a global clinical research platform designed to facilitate clinical research in Huntington’s disease. Core datasets are collected annually from all research participants as part of this multi-center longitudinal observational study. Data are monitored for quality and accuracy using a risk-based monitoring approach. All sites are required to obtain and maintain local ethical approval.

Enroll-HD version PDS5 was used for this study, which originated from the Enroll-HD electronic data capture database on October 31, 2020 at 23:00 UTC. The data is collected from 171 sites across 20 countries and describes baseline and longitudinal variables for 21,116 participants, see Fig. 1a [7].

### Enroll-HD inclusion criteria

Participants with an AAO or rater’s estimated AAO (sxrater) lower than 21 and a CAG repeat size above 59 were excluded to remove juvenile HD patients from the cohort. Also, participants with a CAG repeat size below 36 were excluded. This resulted in a dataset of 11,397 participants, with 36,980 study visits to healthcare facilities, see Fig. 1b. All variables in the cohort are described in Additional file 2 and online in the data dictionary of Enroll-HD [22].

### Pre-processing

The pre-processing of the Enroll-HD dataset is explained in the following sections and shown in Fig. 1c.

#### Filtering variables

Firstly, variables with a high percentage of missing values (> 69%) were excluded from the analysis. Secondly, variables that were redundant were also filtered out. For example, the variable ‘sit‘ indicates whether the stroop test was completed during a visit. However, this can be deduced from the results saved in the variables ‘sit1‘ (total correct), ‘sit2‘ (total errors), and ‘sit3‘ (total self-corrected errors). Thirdly, some variables which have been used to create a derived variable, that represents the data stored in the used variables better, are excluded. For example, the maternal AAO (momagesx) and the paternal AAO (dadagesx) are excluded, because they are used to create the parent’s AAO variable (parentagesx). Fourthly, variables which are considered not to have any valuable information are excluded e.g. the variable ‘xbsp’ which describes if there is additional biosample data available for the participant. Finally, some variables are excluded for other reasons, which require more elaboration, and are therefore explained in later sections. All variables filtered out from the dataset are listed in Additional file 3 with a category and description explaining the reason for filtering.

#### Filtering numerical values

The approach proposed by Cousineau et al. [23] was used to detect numerical errors in continuous variables. This approach detects values that fall outside the mean range ($\mu$) plus four times the standard deviation ($\sigma$). The values detected outside this range were evaluated manually to establish if they indicated an incorrect annotation. All values were found to be correct and were kept. The distribution of the detected variables are shown in Additional file 4.

#### Calculating derived variables

Two variables were created by combining multiple variables. The first derived variable is the “Parent AAO” variable. The Parent AAO records either the maternal AAO (momagesx) or paternal AAO (dadagesx). However, the AAO of the parent with the youngest AAO was used if both were recorded, which was only the case in five participants.

The second derived variable was the CAG age product (CAP) variable, a variable created by Penney et al. [24] and was found to reflect the progression of striatal pathology. The CAP score estimates the disease state of a patient. A CAP score of 100 indicates that the patient has reached the expected AAO of motor symptoms. The score can also be seen as the exposure to the toxic effects of the mutant HTT protein. The CAP score (*C*) was calculated as described by Warner et al. [25]. This score uses the current age (*A*) of the participant, the larger CAG allele repeat length (*R*), and two constants *L* and *K*, which were set to 30 and 6.27 respectively, as proposed by Warner et al., in [25].

$$\begin{aligned} C = A \times (R - L) \times K \end{aligned}$$

#### Inferring missing values

Many missing values represented occasions where values were not recorded for a particular healthcare visit due to three main reasons. Firstly, baseline variables were recorded as missing in the follow-up visits. These are only recorded at the first visit in case of the Medical History variables. Here values from the first visit were used to impute the values in the follow-up visits. Secondly, General Variable Items II variables were only recorded when a variable’s value changed in comparison to the latest visit. Here values from the latest visit were used to impute the values in the follow-up visits. Thirdly, variables not applicable to a participant also contain many missing values. For example, if a participant has never smoked, the variables that record smoking habits, tobcpd (cigarettes pet day) and tobyos (years of smoking), were missing. In such cases, the values of those variables were replaced with a zero. Finally, if a participant’s value for the AAO (hddiagn) was not clinically diagnosed yet, it was replaced by the rater’s estimate of the AAO (sxrater), if available. This was deemed sufficient since the AAO had a significantly high Pearson correlation ($r>0.9; p<.01$) with the estimated AAO ($r=.937$). In addition, the estimated AAO is accompanied by a confidence variable. Here, a high correlation was also found for an unknown ($r=.939$), lower ($r=.916$), or higher ($r=.944$) confidence of estimated AAO, see Additional file 5 for more details.

#### Filtering AAO of symptoms

There were a lot of variables recording age of symptom onset that did not have an estimated value e.g., AAO of motor symptoms (ccmtrage). Each age of symptom onset variables has a related variable which indicates whether the onset of a particular symptom has begun, e.g. ccmtr for the AAO of motor symptoms. If so, the related variable records a one, if not a zero, and if it is unknown a missing value (NaN). All the age of symptom onset variables and associated related variables are shown in Additional file 6. Missing values in the age of symptom onset variables can only be imputed if the related variable recording the symptom onset has the value of one. In all other cases (zero or NaN) no assumptions can be made whether the onset of a symptom has begun. If more than 20% of the related variable record a 0 or NaN the age of symptom and related variable are excluded, since excluding the participant instead would drastically shrink the dataset. Therefore, only the variables parentagesx (Parent AAO), sxsubj (initial symptom noted by patient), sxfam (initial symptom noted by family), ccmtrage (AAO of motor symptoms) are kept. For these variables, participants with a missing value (NaN) are excluded since it is unknown whether the variables should be imputed. The exact percentages of missing values, including the reason why they are missing, are shown in Additional file 7.

#### One-hot encoding

Another important pre-processing step, which is used on all nominal values, is dummy encoding. This encoding transforms a nominal value into *C* boolean variables referring to the presence of the categorical value, where *C* is the number of categories described in the nominal value [26]. All one-hot encoded variables are shown in Additional file 8.

### Imputation of missing values with machine learning

After pre-processing 486 variables were available in the dataset. The remaining missing values were imputed by applying and evaluating ML algorithms for each variable with a missing value. All variables used for imputing are listed in see Additional file 9. To create a training set for variable (*v*) only rows were selected with no missing values in *v*. Any other variables with missing values would be discarded for the imputation of *v*. Variables with continuous values were predicted using regression models and evaluated using the R2 score. Ordinal and nominal variables were clipped and rounded to the appropriate range and categories, and were evaluated using the weighted F1 score.

Models were created for linear regression, logistic regression, random forest, and K-nearest neighbors (Knn), from the sklearn package v1.1.2 [27], and were trained and evaluated using 10-fold cross-validation.

The models were trained using 10-fold cross-validation and the variables were imputed sequentially and ordered by their R2 or F1 scores (highest to lowest). After each imputation of a variable, new models were trained to allow for the addition of more data for the next variables. However, the initial model was used if it outperformed subsequent models. The complete imputation workflow is shown in Fig. 1d.

Composite variables were not imputed but recalculated by the imputed variables that composed them. These were in total 11 variables that included total motor score (motscore), total functional score (tfcscore), functional assessment score (fascore), current packyears i.e., lifetime exposure to tobacco (packy), historic packyears (hxpacky), and the PBA scores: depression (depscore), irritability aggression (irascore), psychosis (psyscore), apathy (aptscore), executive (exfscore), and disoriented behaviour (dbscore).

### Evaluation metrics

Multiple metrics were used to evaluate the ML models for imputation and prediction. To evaluate the classification models for imputation and driving capability predictions, metrics based on the true positive (TP), false positive (FP), true negative (TN), and false negative (FN) were used. These include the accuracy, F1 score, and the area under the receiver operating curve (AUROC) or area under the curve (AUC) for short. Evaluation of regression models for imputation and AAO prediction are based on metrics using the predicted outcome ($\hat{y}$) and the actual outcome (*y*). These include the mean absolute error (MAE), root mean squared error (RMSE), and the R2 score.

$$\begin{aligned} Accuracy= \;& {} \frac{TP + TN}{TP + TN + FP + FN} \\ F1\ score= \;& {} \frac{2TP}{2TP + FP + FN} \\ MAE=\; & {} \frac{\sum ^{n}_{i=1}{ |y_i - \hat{y_i}| }}{n} \\ RMSE= \;& {} \sqrt{\frac{\sum ^{n}_{i=1}{ (y_i - \hat{y_i})^2 }}{n}} \\ R2\ score=\; & {} 1 - \frac{ \sum ^{n}_{i=1}{ (y_i - \hat{y_i})^2 } }{ \sum ^{n}_{i=1}{ (y_i - \bar{y})^2 } } \end{aligned}$$

### Predicting AAO

The performance of the Langbehn formula to predict the AAO was compared to linear regression, linear support-vector machine (SVM), RF, Knn, multi layer perceptron (MLP) [27], eXtreme gradient boosting (XGBoost) v1.5.1 [28], CatBoost v1.0.6 [29] and Light Gradient Boosting Machine (LGBM) v3.3.2 models [30]. These ML models were selected to see whether AAO was better predicted by linear or non-linear patterns and/or different strategies. All models were trained and evaluated using 10-fold cross-validation. The variables used to train the ML models are also listed in Additional file 10 and include the smaller and larger CAG allele repeat size (caglow & caghigh), gender (sex), parent AAO (parentagesx), and whether there was a family history for HD (fhx).

The Langbehn formula (see equation below) was initially developed for predicting the AAO of patients with a CAG repeat range of 41–56. However, our dataset included a wider range of CAG repeats of 36–59. Therefore all ML algorithms were fitted on participants with a wider CAG repeat range of 36–59 and on participants with a narrower CAG range of 41–56 to be consistent with the initial development of the Langbehn formula.

Aside from evaluating the Langbehn formula, the formula was also refitted on the Enroll-HD dataset to draw a more direct comparison with the ML models. The following Langbehn formulas were evaluated:

- The Langbehn formula evaluated on the imputed dataset using the narrow CAG repeat size range (41–56), see equation below.
- Langbehn formula refitted on the imputed dataset using the narrow CAG repeat size range (41–56).
- Langbehn formula refitted on the imputed dataset using the wider CAG repeat size range 36–59.

$$\begin{aligned} Langbehn = 21.54 + \exp {(9.556 - (0.146 \times CAG))} \end{aligned}$$

All the models described above were trained and evaluated only on participants with a clinically diagnosed AAO. For example, participants in which the clinically diagnosed AAO was missing and therefore it was replaced with the rater’s estimated AAO (sxrater) were discarded. The refitted Langbehn formula’s weights were set according to the initial weights of the Langbehn formula and were then fitted using the curve fit function from Scipy v1.9.0 [31]. Finally, the refitted Langbehn formulas were fitted on the whole dataset. The workflow for predicting the AAO is shown in Fig. 1e.

### Predicting driving capability

A RNN with gated recurrent units (GRUs), as described in Cho et al. [32] was used, to predict driving capability longitudinally. The model was trained using 141 variables, listed in Additional file 10, to predict for each visit the current driving capability i.e., when the participant is at the clinic and the driving capability of the participant for the visit in the next year.

The data was reshaped to train the RNN for longitudinal predictions. Participants had between 1 and 5 visits recorded in the dataset, approximately one year apart. Consequently, the data was reshaped to a maximum of five visits that are one year apart. This was achieved by calculating the index of each existing visit using the equation below, where $v_{pi}$ is the new visit index of visit *i* for participant *p*, *d* is the number of days from the baseline visit (visdy), and *t* is the desired number of days between each visit. Here *t* is set to 365 to ensure that all participants have the first visit and that subsequent visits are around one year apart.

$$v_{pi} = \lfloor (d_{pi} - d_{p0}) / t \rceil$$

The remaining missing visits were masked by filling the visit vector with an arbitrary mask value, which the RNN detects and skips using a masking layer, making training faster.

The holdout strategy was used to create the subsets to train and test the model. Here the dataset was split into two by randomly selecting 80% of participants for the training set, with the remaining 20% assigned to the test set. The split was done on groups which were defined by the number of missing visits for each participant. This ensured that the training and test sets had a similar number of missing visits per participant. Continuous input variables were scaled to a range of − 1 to 1 using the MinMaxScaler from the sklearn package [27].

During training, class weights were used to account for imbalance in the label distribution. The weight of a class ($w_c$) was calculated based on the total samples (*n*) divided by the number of unique classes (*C*) times the number of samples of a class ($n_c$), excluding imputed labels, see equation below.

$$w_c = \frac{n}{C \times n_c}$$

In addition, sample weights were calculated to account for missing labels which could be caused by missing visits or visits with imputed labels. In such cases, the sample weight was set to 0. All other sample weights were set to the associated class weight.

The RNN was tuned with a learning rate of 1e−5 using three and five GRU layers with a hidden size of 128, 256, and 512. In addition, three different L2 regularization values were tested, namely 1e−7, 1e−5, and 1e−3. The workflow of predicting driving capability is shown in Fig. 1e.

## Results

### Pre-processing

**Fig. 2:**
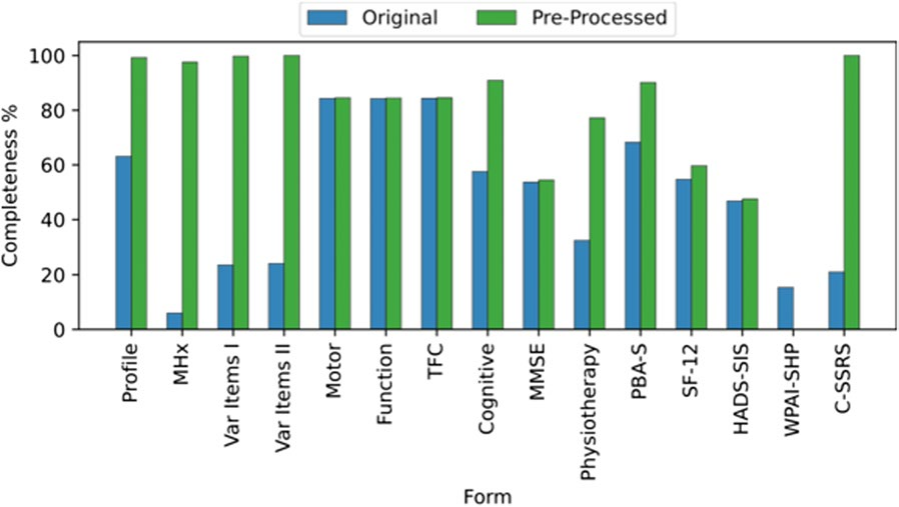
The completeness percentage of each form in the Enroll-HD dataset before and after pre-processing. Here the blue bars represents the completeness of the original Enroll-HD data. The green bars represent the completeness of the data after pre-processing, see Fig. 1a

Pre-processing of the cohort data resulted in a significant increase in completeness of all variables in the cohort, from 48.56 to 93.2%. Here pre-processing included: filtering variables which are redundant or have a high percentage of missing values, filtering numerical values, calculating derived variables, inferring missing values, filtering AAO of symptoms which could not be imputed, and one-hot encoding, see Fig. 1c. The completeness of the dataset, before and after pre-processing, is demonstrated in Fig. 2. The figure shows the completeness per form, where a form describes multiple variables related to each other. There were three strategies that we applied in order to improve the completeness per form, by reducing the amount of missing data. The forms that benefited the most from these strategies were MHX, Var Items I, Var Items II and C-SSRS. Firstly, forms MHX and Var Items II mostly contained missing values in the follow-up visit which could be inferred from the baseline visit or latest visit respectively. Secondly, the form Var Items I contained many categorical variables which are transformed using one-hot dummy encoding or because some variables could be inferred from other variables. Thirdly, the form C-SSRS contained many variables above the missing value threshold of 69%, which were excluded. The form which was reduced in completeness is the WPAI-SHP form, because it mostly contained variables with 90% missing values, which were excluded.

### Imputation performance

**Fig. 3:**
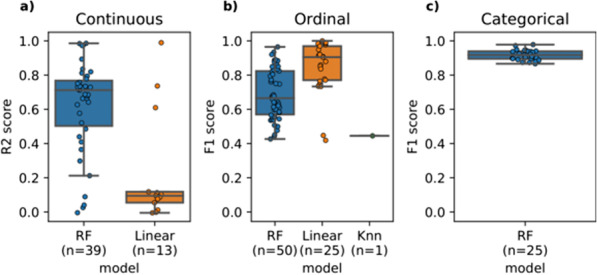
The mean R2 and F1 scores of the best performing imputation models. This is shown for each variable in each data type, namely **a** continuous, **b** ordinal, and **c** categorical. The trained models were: random forest (RF), linear/logistic regression (Linear), and K-nearest neighbours (Knn)

After pre-processing we used three models to impute the remaining 6.8% missing values. For each type of variable (continuous, ordinal, and categorical) the models RF, linear regression and Knn were evaluated. The performance of the best imputation model per variable is shown in Fig. 3 depicted as R2-scores or F1-scores (highest mean R2 or F1 score). All the scores are visualized in Fig. 3 and their corresponding best model and best round of imputation are available in Additional file 11. RF performed the best to impute the variables on 114 occasions and linear regression on 38 occasions. Knn on the other hand performed the worst, imputing only one variable.

Continuous variables were generally predicted with an R2 score greater than 0.6 (Fig. 3a). Some continuous variables were predicted with an R2 score between 0 and 0.6. 81% of ordinal and categorical variables were predicted with an F1 score of 0.6 or greater, see Fig. 3b and c. However, there were also some (19) ordinal variables predicted with a lower F1 score (0.4–0.6).

### AAO prediction

To predict the AAO, we used several ML models and compared them to the Langbehn formula. We compared the models on two subsets, one subset which only included participants with a narrow range of CAG repeats (41–56) and another subset which included a wider CAG repeat range (36–59), encompassing all participants in our cohort. Firstly, the models were trained on participants with a narrower CAG repeat size (41–56), since the Langbehn formula was developed to predict the AAO of participants within that range. The performance of all models on the narrower CAG repeat size, indicated by an “n”, is shown in Table 1. In this ‘n’ range we observe that the refitted Langbehn formula outperformed the original model, which was also outperformed by the majority of the ML models (CatBoost,LGBM, XGBoost, MLP, linear regression). The LGBM model performed the best with an R2 of 0.60, MAE of 5.26 and RMSE of 6.87 indicating that this model can improve the estimation of AAO predictions while keeping the error margin lower than any of the models tested.

Secondly, we tested the aforementioned models on the participants with a wider CAG range (36–59), indicated by a ‘w’, see Table 1. As previously, the LGBM model performed the best among all the models with a R2 score of 0.63, a MAE of 5.46 and RMSE of 7.16. Although, a direct comparison between the different subsets ‘n’ and ‘w’ cannot be made, we do however observe that the R2 of the LGBM model was higher but the MAE and RMSE were slightly increased.

In summary, the ML model LGBM outperformed the Langbehn formula, in both cases with the narrower and wider CAG repeat range. In addition, the LGBM model not only had a better performance but achieved a lower error in both cases as well, than the Langbehn formula. This shows that the LGBM model has a better performance and works for a broader CAG repeat range compared to the Langbehn formula.

**Table 1:** The performance of the Langbehn formulas and the ML models to predict the AAO using participants with a narrow (n: 41–56) and wider (w: 36–59) CAG repeat size (caghigh)

Model	MAE		RMSE		R2	
	n	w	n	w	n	w
LGBM	5.26	5.46	6.87	7.16	0.60	0.63
CatBoost	5.29	5.49	6.91	7.20	0.60	0.62
Linear regression	5.45	5.77	7.07	7.50	0.58	0.59
Langbehn refitted	5.38	5.74	7.09	7.57	0.58	0.58
MLP	5.46	5.77	7.14	7.55	0.57	0.58
XGBoost	5.48	5.65	7.15	7.41	0.57	0.60
Langbehn	5.57	5.91	7.23	7.88	0.56	0.55
Linear SVM	5.68	5.96	7.35	7.75	0.55	0.56
Random forest	5.85	6.03	7.63	7.89	0.51	0.55
Knn	6.18	6.37	8.11	8.37	0.45	0.49

### Model performance driving capability

The longitudinal nature of Enroll-HD can be exploited by ML models that can learn temporal dynamic behaviour. We used a RNN with GRUs to predict the driving capability for the coming and next year for each time-step available per individual participant. In total 18 combinations of hyperparameters were tested to predict driving capability. The model with the highest AUC and F1 on the test set resulted in an AUC of 0.929, an accuracy of 0.852, and a F1 score of 0.819. The evaluation metrics and the hyperparameters for all the models are shown in Additional file 12.

**Fig. 4:**
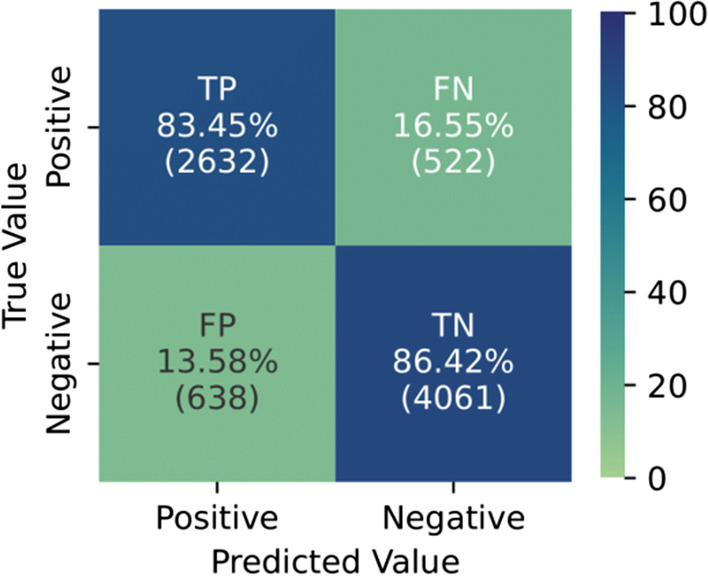
The error matrix for predicting driving capability in the test set. Here the percentages refer to the percentage of positive samples (able to drive) predicted as a positive (TP) and the percentage of positive samples predicted as a negative (TN). The same is true for the negative samples (unable to drive)

The error matrix of the best model is shown in Fig. 4. 83.45% of the positive samples (1: status is driving) are predicted as positive and 86.42% of the negative samples (0: status is not driving) are predicted as negative. Indicating that the GRU model is better at predicting negative samples, possibly due to the higher number of training samples for the negative label in the dataset.

#### Personalized predictions for driving capability

**Fig. 5:**
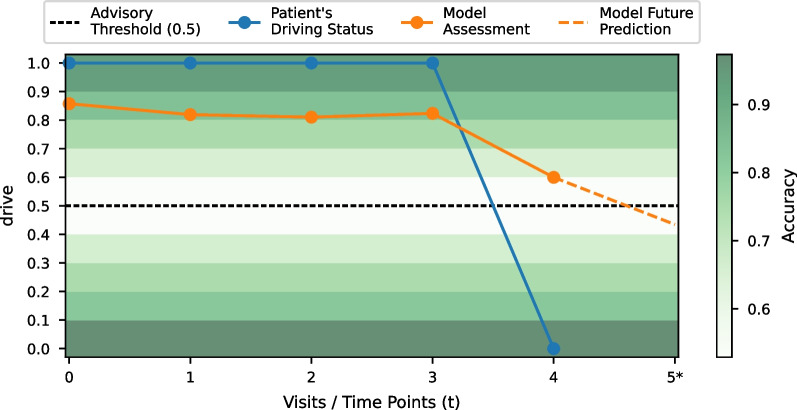
An example of a personalized prediction of the driving capability of an Enroll-HD participant. The blue line indicates the clinical assessment and the orange line indicates the assessment of the ML model. The dashed orange line indicates a prediction over a future time point (time point 5) for this particular individual. The two distributions, clinical assessment and model assessment, do not align at time point 4, which indicates that a patient might have been wrongly assessed by the clinic at that time point, as the model predicted a value around 0.6

We applied the GRU model to perform predictions for each participant at each time step. An example of a prediction on a single participant is shown in Fig. 5. The recorded driving status in Enroll-HD in this plot is binary (blue line), either zero or one, and the predicted driving status (orange line) by our model can range from zero to one. A value above the threshold of 0.5 (dashed black line) corresponds to a positive advice to drive and a value below the threshold corresponds to a negative advice to drive. For every prediction an accuracy score is provided in the range of zero to one in ten bins with a step size of 0.1. These bins are depicted as different rows/bands in the heat map of Fig. 5. The boundaries of these bands are depicted by the tick marks on the y-axis. The accuracy score is calculated by taking all samples from the test set that were predicted within that particular band and calculating the accuracy for the predicted samples within that band. This results in an accuracy score for each band to make the prediction more interpretable. The accuracy of the predicted value is higher, around 90%, when it is near zero or one, and less accurate, around 60%, when it is near the threshold.

The personalized prediction in Fig. 5 shows that in time step/visit 4 the model’s assessment (orange line) is different from the participant’s driving status (blue line), recorded in the Enroll-HD dataset. This indicates that a participant could possibly still drive at that time point. However, the prediction accuracy is near the advisory threshold (dashed black line), which may indicate either a miss-classification or an incorrect assessment. Either way, this points to further exploration of the driving skills of this participant.

In addition, our model provides a future prediction (dashed orange line) for the driving capability, by learning from data from previous time points. The future prediction is shown as the fifth time point in Fig. 5. Here the model predicts that the participant is advised not to drive anymore in the next year.

**Fig. 6:**
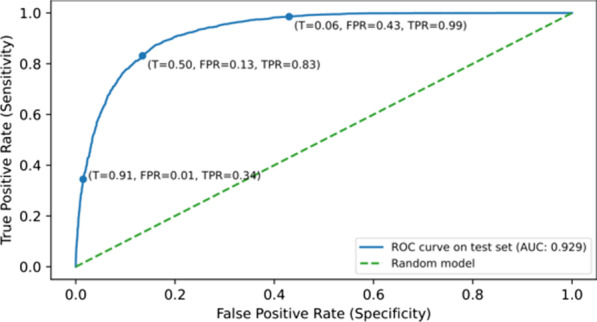
The ROC curve calculated on the test set for the driving capability. The ROC curve (blue line) is shown with a simulated random model (dashed green line) and multiple advisory thresholds (T), indicated by the blue dots. The thresholds 0.91 and 0.06 show that a predicted value above 0.91 and below 0.06 results in a correct prediction for the positive class and negative class respectively in 99% of the test cases. The default threshold of 0.5 shows that an AUC of 0.929 is achieved and a TPR of 0.84 and FPR of 0.13

The ROC curve in Fig. 6 shows the model’s performance on the test data. The figure shows that the ROC curve (blue line) is above the simulated random model (dashed green line). This indicates that the model is correctly distinguishing the negative class (unable to drive) from the positive class (able to drive). The default threshold is set to 0.5, which results in a true negative rate (TNR), the rate at which the model correctly identifies participants not able to drive, of 0.87 (TNR = 1 − FPR). In addition, the model achieves a true positive rate (TPR), the rate at which the model correctly identifies participants able to drive, of 0.83. Finally, an AUC of 0.929 is achieved which indicates that the model can almost perfectly seperate the two classes using this threshold. However, when the threshold is set to 0.06 the TPR becomes 0.99. This shows that a predicted driving capability below 0.06 is very likely to be actually negative. On the other hand, the threshold can be set to 0.91. This results in a TNR of 0.99, which indicates that a predicted driving capability above 0.91 is very likely to be actually positive.

## Discussion

In this paper we show the utility of applying ML models to pre-process and analyse the extensive, longitudinal data collection from Enroll-HD. Our models outperformed a leading prediction approach (Langbehn formula) for the estimation of the AAO. We also made use of the longitudinal information through GRUs to assess driving capability and accurately predict future time points. We were able to make these improvements through the application of simple pre-processing methods and further ML imputation, maximising the number of participants and variables included in our study. In addition, we provided a complete list of the variables included in this analysis, details on pre-processing steps of all variables and a workflow deposited in an online repository to increase the reproducibility of our results and the reusability of our workflow.

### Pre-processing and imputation

In order to reduce missing values, the selected cohort was pre-processed, increasing the completeness of the dataset from 48.56 to 93.2%. This was a crucial step, as ML algorithms need complete data to make predictions. In this process we found that most missing values do not necessarily indicate an unknown value and could be inferred by other variables in the dataset.

The increase in completeness of the data can partially be attributed to the excluded variables, listed in Additional file 3. However, some of these excluded variables might have been relevant for either the AAO or driving capability prediction. For the AAO model, the latest study site region of the participant’s visit (region) variable could possibly reveal additional information. For example, it could provide insights into biases of the assessments of AAO in the different regions. For the driving capability model the excluded AAO of symptoms could have possibly been predictive. These include onset of psychosis (ccpsyage), onset of aggressive behaviour (ccvabage), onset of perseverative obsessive behaviour (ccpobage), onset of apathy (ccaptage), onset of irritability (ccirbage, and onset of depression (ccdepage). These variables would indicate the beginning of decline in a specific symptom, which could indicate a future decline in driving capability, and therefore improve the prediction accuracy. In the future machine learning could be applied on a subset of Enroll-HD participants which have complete data on the assessments regarding the AAO of the symptoms. It would be interesting to investigate whether the inclusion of these variables has an impact on the model’s performance.

After pre-processing the remaining 6.8% missing values were imputed using ML models. Most of the variables could be imputed with a relatively high accuracy score, but also those with a lower score we decided to impute them nonetheless. The continuous variables were kept, since the ML models outperformed the more traditional imputation approach by imputing variables with the mean. This was the case, since all variables were imputed with a R2 score above 0. Regarding the ordinal and categorical variables, these were associated with a very small fraction of the imputed variables (up to 17% out of the 6.8% of the missing values), lowering the risk to influence the predictive performance of our model. Also, keeping these variables increases the number of predictive input variables and visits in the dataset.

The current imputation models (RF, linear/logistic regression, and Knn) were chosen because of their low computational cost, as the current imputation involved many (167) variables. However, future studies should consider testing and evaluating a wider variety of ML models for imputation, in terms of complexity, in order to improve the accuracy of the imputation.

### Using ML to predict AAO

The Langbehn formula, developed for the most frequently occurring CAG repeat lengths (41–56) has been the best available model for AAO prediction. However, we were able to improve on this by using multivariate data and more complex ML models to find more predictive patterns. In addition, we also used the full distribution of the participants’ CAG repeats (36–59) that was recorded in the Enroll-HD data, to test the broader applicability of the Langbehn formula and to evaluate the performance of the ML models on this CAG repeat size range.

A more precise AAO estimation could support upcoming clinical studies that develop therapies that can modify or delay the AAO by selecting the right HD population, without unnecessarily including participants too early or too late. The LGBM model showed the largest improvement in AAO estimation for both the narrow and wider CAG repeat range. Although the wider CAG range is not as common, it is still an HD causing CAG range, and is therefore relevant. We demonstrated that a better performance could be achieved by including more information, so we speculate that future studies, including both observational and genetic information from pre-manifest patients, would lead to more accurate AAO estimations.

Overall, the difference in error and performance between the ML models and the Langbehn formula indicate that analysing large datasets, such as Enroll-HD, with more complex ML models and adding only a few more variables can provide better estimations of the clinical AAO.

### Using ML to advise driving capability

Clinicians involved in the care of HD patients are often asked for advice regarding functional capabilities, based on clinical characteristics. Such advice is, for example, the ability for a patient to drive their car after symptoms onset. Assessing this adequately can impact the patient’s quality of everyday life and in some countries can result in revocation of a driver’s license. However, this is difficult to assess since HD involves many different symptoms; motor, cognitive, and behavioural, which change over time and can all influence driving capability.

To assist clinicians in this task, we created an advisory model. This model complements the clinician’s experience-based decision-making with a data-driven assessment of driving capability. Our model can be beneficial in cases where the clinician is uncertain of a HD patient’s driving capabilities. The model provides the clinician with a prediction of driving capability for the coming five years based on 142 clinical characteristics, see Fig. 5. Therefore, our driving capability model is a tool to provide additional information to help clinical decision-making processes. However, it is not designed to replace decision-making on driving capability. In some cases, additional assessments are necessary to determine driving capability. For instance, an examination by an occupational therapist or on- and off-road driving assessments such as the DriveSafe DriveAware app [33]. This app measures the driver’s awareness of the environment and awareness of the patient’s driving ability to predict if the patient requires an on-road assessment.

Aside from current driving capability, our model provides a trajectory of the driving capability one year ahead serving as a prognostic tool to further support driving capability assessment. The trajectory can give an indication to the patient that even though it still might be safe to drive, the situation is likely to change in the coming year. With this information the patient could take precautions and take decisions regarding their every-day life.

Our advisory model provides a prediction value between 0 and 1 together with an accuracy score. This combination means that when a prediction value is close to the classification threshold (0.5) it corresponds to a low accuracy score of around 0.5–0.7, which indicates that it might be unsafe for the participant to drive.

It is important to note that our advisory model for driving capability is not a generalized model, but a model that is tailored to the Enroll-HD study. For example, any newly enrolled participant in Enroll-HD could get a prediction value for their driving capability. Other HD cohorts could build on our workflow to pre-process their data and train their own model. If possible, cohorts can be combined to train the model on more data, which could improve the model’s accuracy.

In the future, the model’s predictive performance could be increased by evaluating the predictive power of the variables, with for example the SHAP algorithm [34]. Using this information, only the most predictive variables would be included. Thus, reducing the complexity this would also make the model more interpretable.

Another important note is that our advisory model is trained to predict the driving capability based on the driving status of the participant, instead of their actual capability. This means that the driving prediction can be influenced by how the HD patient assesses their own driving capability or how their family assesses them, which the model cannot take into account. This could affect the model to either prioritize the quality of life of the patient, by giving a more positive advice, or the safety for other road users, by giving a more negative advice. If necessary the classification threshold (Fig. 5 can be tuned to prioritize a positive or negative advice.

In the future, this work can be extended by predicting symptom trajectories and many other daily living activities that patients need to be advised on.

## Conclusions

ML is a promising approach for analyzing large heterogeneous observational data such as Enroll-HD. The application of ML for data pre-processing led to a significant reduction of missing values in the dataset, increasing its completeness by including more variables and participants. The pre-processing workflow is available to the public and was extensively described to increase reproducibility and adoption of ML in Enroll-HD. ML models achieved a higher performance, while maintaining a lower error rate, in estimating the AAO of Enroll-HD participants in comparison to the Langbehn formula. Finally, ML was also applied to create a model to assist clinicians in advising and providing a future trajectory regarding the driving capability for HD patients. ML might be a promising approach to facilitate personalized patient care for assisting with daily activity assessment and symptom trajectory prediction.

## Supplementary Information

**Additional file 1**: Workflow: A figure of the complete workflow of the methods. a) Shows the cohort selection, inclusion criteria, and pre-processing steps. b) Shows how the ML models are trained to impute the missing values. c) Shows how the imputed dataset is used to fit and evaluate the ML models and the Langbehn formula for the AAO prediction. d) Shows how the imputed dataset is used to fit and evaluate the ML models to predict the driving capability.

**Additional file 2**: All Variables: A table describing all the variables in the cohort, including the form belonging to the variable, the data type, and a description of the variable.

**Additional file 3**: Excluded Variables: A table of the excluded variables and their percentage of missing values with a category describing why the variable has been excluded.

**Additional file 4**: Detected outliers: A figure of the detected numerical errors within the selected cohort. Each red bar indicates the threshold of a potential error being detected.

**Additional file 5**: Pearson correlation values between the AAO, larger CAG allele repeat size, and AAO estimations: A figure of a heatmap with correlation and P-values of the larger CAG allele repeat size, the AAO, the rater’s estimated AAO, and the rater’s estimated AAO with a high, low and missing confidence.

**Additional file 6**: Related variables for age features: A table of the age features and their related variables used to filter the AAO of symptoms.

**Additional file 7**: Missing value percentages for age features: A table of the missing percentages of the AAO of symptoms variables and the reason why they are missing.

**Additional file 8**: The one-hot encoded variables: A table listing the variables transformed and created using one-hot encoding.

**Additional file 9**: Variables used during imputing: A table listing the variables used during imputing.

**Additional file 10**: The pre-processed and input variables for the ML models: A table listing the remaining variables after pre-processing and the input variables for the AAO and driving capability prediction.

**Additional file 11**: The best imputation model for each imputed variable: A table listing the best model and round for each imputed variable, including the R2 or F1 score depending on the variable type.

**Additional file 12**: Evaluation metrics of all the tuned models: A table with all the tuned driving capability GRUs and their evaluation metrics, sorted by the AUC, F1 score, and accuracy for the test set.

## Data Availability

The dataset supporting the conclusions of this article is available at: https://enroll-hd.org/for-researchers/access-data. To access the data you must be a researcher employed by a recognized academic institution, company or nonprofit organization and apply for an Enroll-HD access account. The source code is freely available at https://github.com/JasperO98/hdml/tree/main and an archived version at 10.5281/zenodo.7620222.

## Abbreviations

HD: Huntington’s disease
ML: Machine learning
AAO: Age at onset
HTT: Huntingtin protein
RF: Random forest
PDS5: Periodic dataset 5
RNN: Recurrent neural network
CAP: CAG age product
Knn: K-nearest neighbours
TP: True positives
FP: False positives
TN: True negatives
FN: False negative
AUROC: Area under the receiver operating curve
AUC: Area under the curve
MAE: Mean absolute error
RMSE: Root mean squared error
SVM: Support-vector machine
MLP: Multi layer perceptron
XGBoost: EXtreme gradient boosting
LGBM: Light gradient boosting machine
GRU: Gated recurrent unit
TNR: True negative rate
FPR: False positive rate
FNR: False negative rate
TPR: True positive rate
