# Supplementary figures and images for: Machine learning in Huntington’s disease: exploring the Enroll-HD dataset for prognosis and driving capability prediction
Source: Orphanet J Rare Dis. 2023 Jul 27;18:218. doi: 10.1186/s13023-023-02785-4 (PMC10375780; doi:10.1186/s13023-023-02785-4)

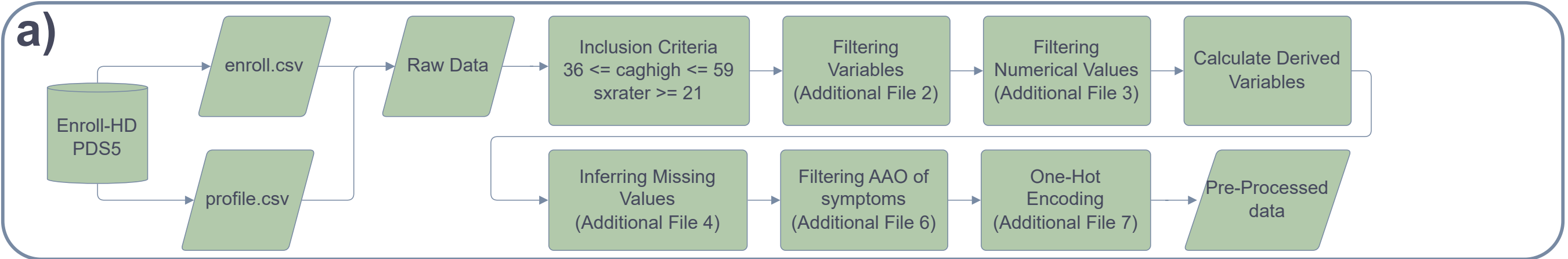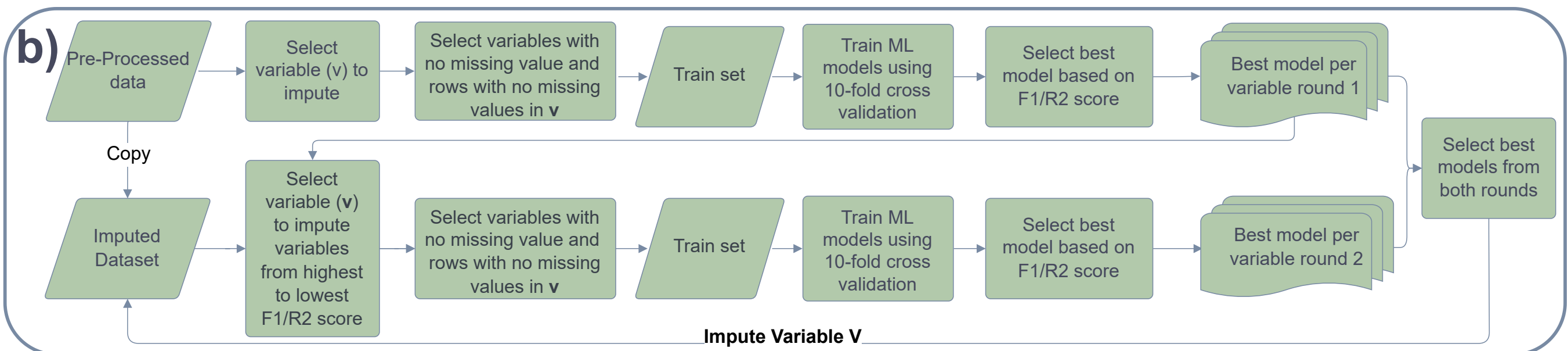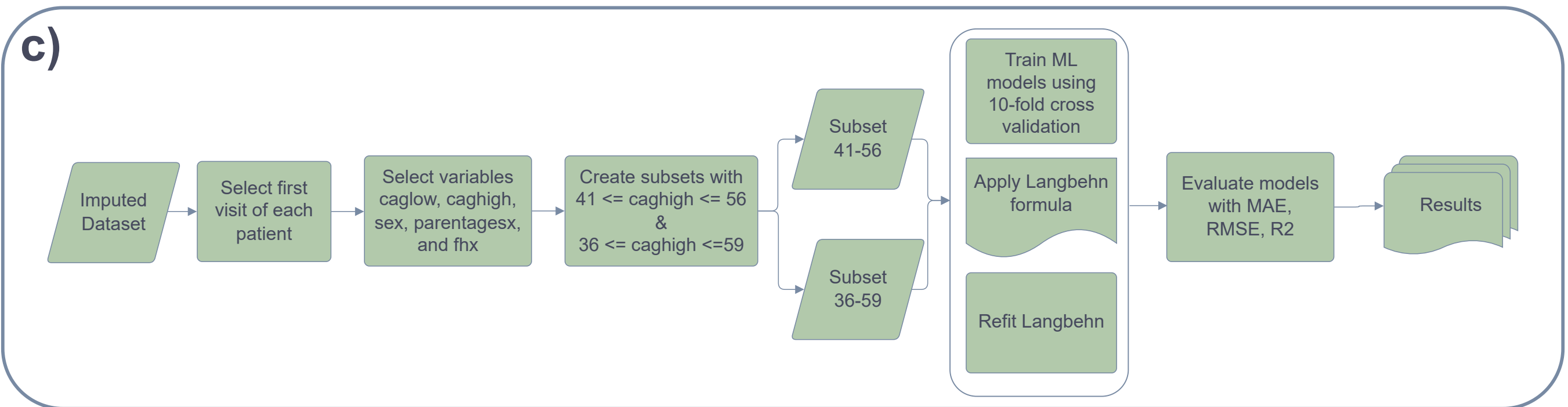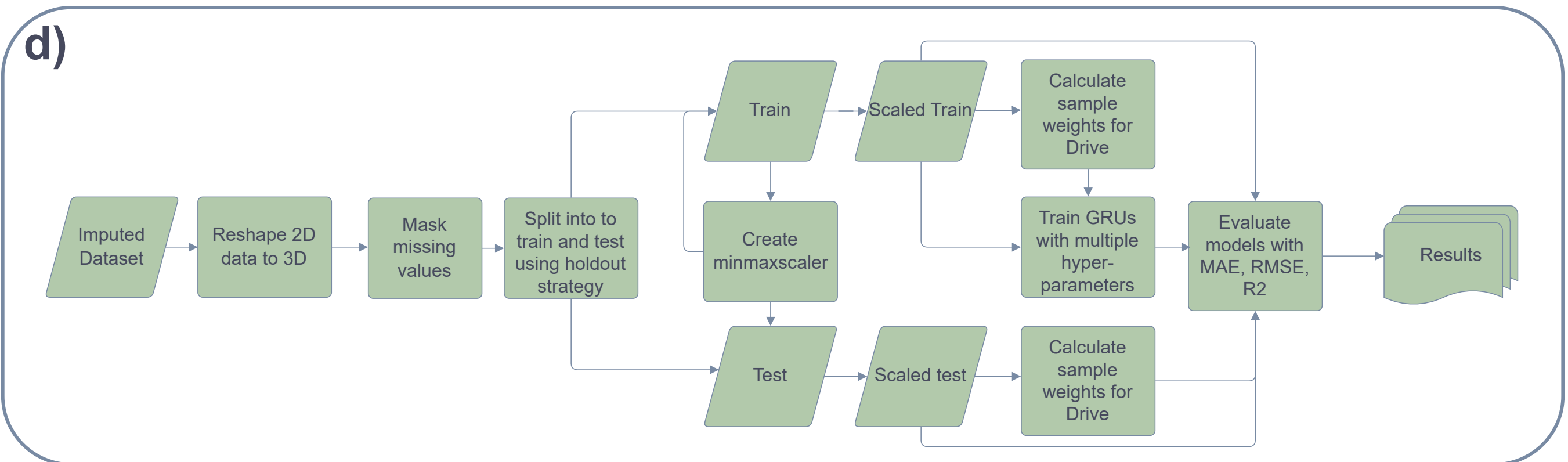

Supplement: Supplementary file 1 — Additional file 1: Workflow: A figure of the complete workflow of the methods. a) Shows the cohort selection, inclusion criteria, and pre-processing steps. b) Shows how the ML models are trained to impute the missing values. c) Shows how the imputed dataset is used to fit and evaluate the ML models and the Langbehn formula for the AAO prediction. d) Shows how the imputed dataset is used to fit and evaluate the ML models to predict the driving capability. [file 13023_2023_2785_MOESM1_ESM.pdf]

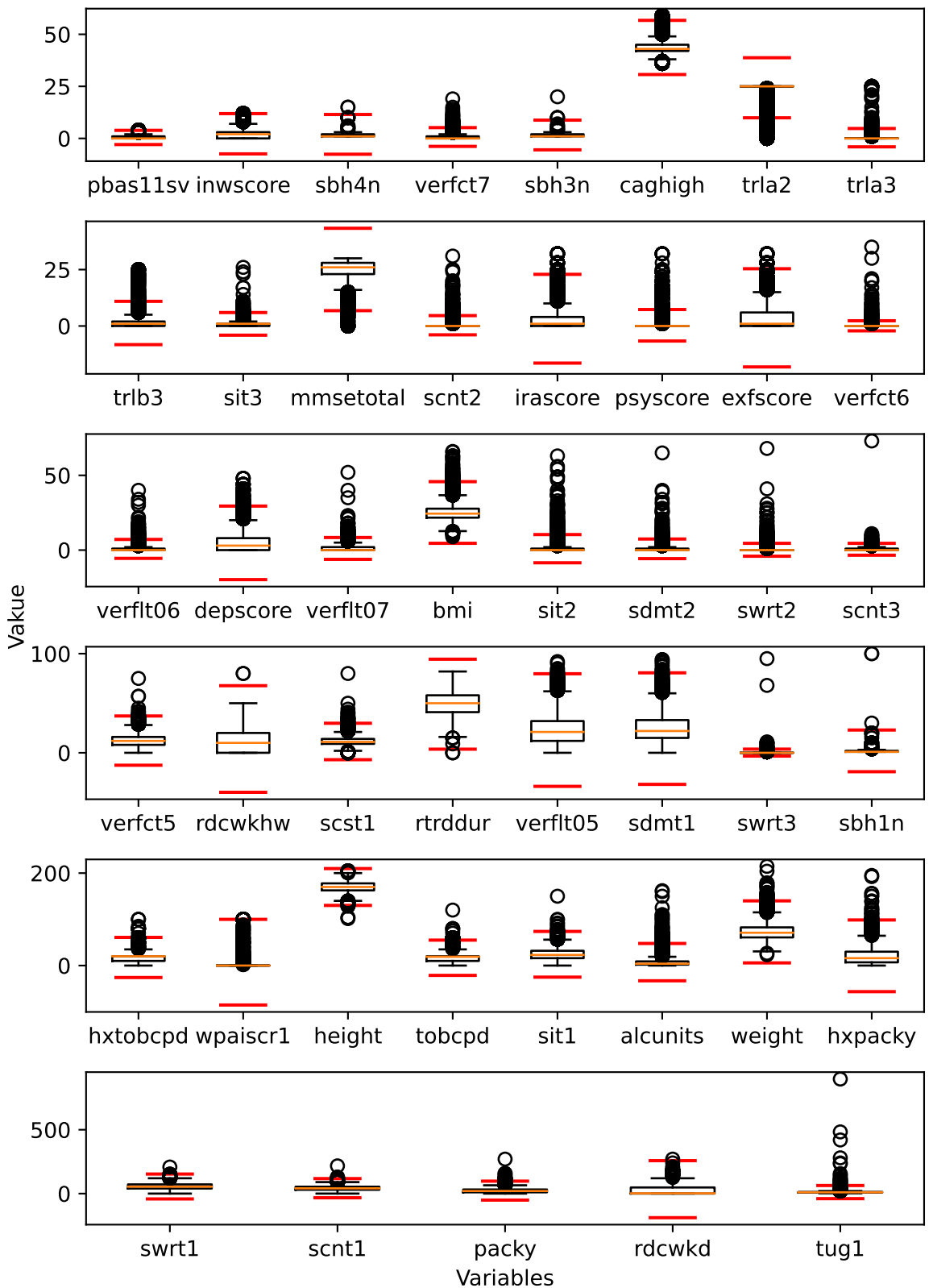

Supplement: Supplementary file 4 — Additional file 4: Detected outliers: A figure of the detected numerical errors within the selected cohort. Each red bar indicates the threshold of a potential error being detected. [file 13023_2023_2785_MOESM4_ESM.pdf]

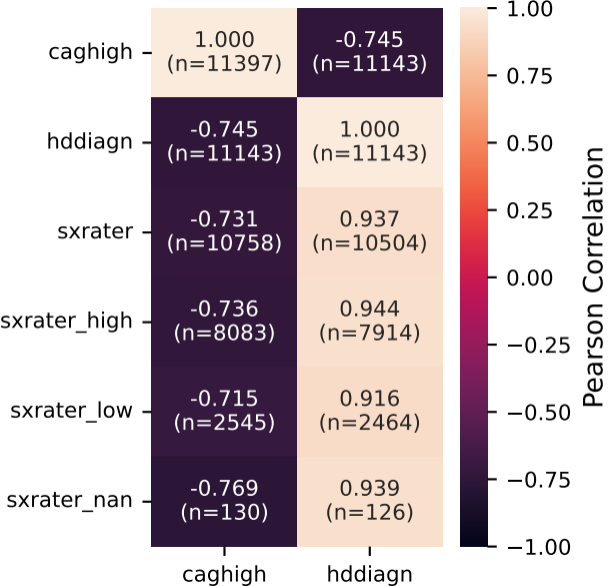

Supplement: Supplementary file 5 — Additional file 5: Pearson correlation values between the AAO, larger CAG allele repeat size, and AAO estimations: A figure of a heatmap with correlation and P-values of the larger CAG allele repeat size, the AAO, the rater’s estimated AAO, and the rater’s estimated AAO with a high, low and missing confidence. [file 13023_2023_2785_MOESM5_ESM.pdf]
